# Dynamics of mitochondrial inheritance in the evolution of binary mating types and two sexes

**DOI:** 10.1098/rspb.2013.1920

**Published:** 2013-10-22

**Authors:** Zena Hadjivasiliou, Nick Lane, Robert M. Seymour, Andrew Pomiankowski

**Affiliations:** 1 CoMPLEX, University College London, Gower Street, London W1E 6BT, UK; 2 Department of Genetics, Evolution and Environment, University College London, Gower Street, London W1E 6BT, UK; 3 Department of Mathematics, University College London, Gower Street, London W1E 6BT, UK

**Keywords:** mating types, mitochondria, mitonuclear coadaptation, selfish conflict, sexes, uniparental inheritance

## Abstract

The uniparental inheritance (UPI) of mitochondria is thought to explain the evolution of two mating types or even true sexes with anisogametes. However, the exact role of UPI is not clearly understood. Here, we develop a new model, which considers the spread of UPI mutants within a biparental inheritance (BPI) population. Our model explicitly considers mitochondrial mutation and selection in parallel with the spread of UPI mutants and self-incompatible mating types. In line with earlier work, we find that UPI improves fitness under mitochondrial mutation accumulation, selfish conflict and mitonuclear coadaptation. However, we find that as UPI increases in the population its relative fitness advantage diminishes in a frequency-dependent manner. The fitness benefits of UPI ‘leak’ into the biparentally reproducing part of the population through successive matings, limiting the spread of UPI. Critically, while this process favours some degree of UPI, it neither leads to the establishment of linked mating types nor the collapse of multiple mating types to two. Only when two mating types exist beforehand can associated UPI mutants spread to fixation under the pressure of high mitochondrial mutation rate, large mitochondrial population size and selfish mutants. Variation in these parameters could account for the range of UPI actually observed in nature, from strict UPI in some *Chlamydomonas* species to BPI in yeast. We conclude that UPI of mitochondria alone is unlikely to have driven the evolution of two mating types in unicellular eukaryotes.

## 1. Introduction

The existence of two distinct sexes in the majority of sexual organisms poses a well-known conundrum in evolutionary biology [1]. Two sexes would seem to be the worst of all possible worlds, as individuals can only mate with half the population. Either one sex or multiple sexes should be better. A prominent explanation relates to mitochondrial inheritance. Uniparental inheritance (UPI) of mitochondria (or more specifically, mtDNA) is nearly universal among multicellular animals and plants, where one sex (usually the female) passes on its mitochondria, whereas the other does not [2]. UPI is also widespread in fungi, algae and isogamous unicellular organisms with morphologically identical gametes, which nonetheless often correspond to two mating types [3,4].

Why UPI is so widespread is uncertain. There are three main hypotheses. First, UPI may purge deleterious mitochondrial mutations. Specifically, it has been shown that UPI increases the variance in mtDNA between cells, facilitating selection against deleterious mitochondria [5,6]. By contrast, biparental inheritance (BPI) averages the number of mutant mitochondria of parents, hindering selection for the lower mutation load. However, there is no explicit evolutionary model of the transition from BPI to UPI.

A second hypothesis proposes that UPI minimizes selfish conflict between mitochondria and the host cell. Mixing mitochondria (or other cytoplasmic elements) from different parents may select for mutations that are beneficial for the mitochondria (providing a replicative advantage) but harmful to the host cell [7–9]. This hypothesis has received the greatest attention. Evolutionary models using a fixed cost for cells carrying selfish mutants have shown that uniparental mutants are favoured in a biparental population [10] and under specific conditions can lead to the evolution of two mating types [11,12]. The assumption of a fixed cost for BPI in these models is dubious, however, and needs to be further investigated as fitness reduction is expected to depend on the number of selfish mutations carried. Such consideration was made in an earlier analysis by Hastings [13], who concluded that the spread of selfish mutants led to the evolution of UPI and anisogamy. However, this model actually showed that only moderate levels of UPI evolve; and it did not consider the possible invasion of associated mating types.

The third hypothesis relates to our earlier work demonstrating that mitonuclear coadaptation improves with UPI under a wide range of conditions [14]. This hypothesis is based on the fact that oxidative phosphorylation requires multiple interactions between proteins, RNA and DNA encoded in the nucleus and mitochondria [15]. Strong evidence across many eukaryotic organisms demonstrates that the two genomes have indeed adapted to each other over evolutionary time [16–18]. Theoretical work again supports an advantage to UPI [14] but also lacks formal consideration of the evolution transition from BPI to UPI.

These limitations and different models make it difficult to determine the conditions under which the fitness benefits of UPI are sufficient to drive the evolution of two distinct mating types in unicellular organisms. Here, we assess this evolutionary question for all three hypotheses by developing a novel, more extensive model. We explicitly define a nuclear mechanism of mitochondrial inheritance and ask whether UPI could evolve in an ancestral population where mitochondrial inheritance is biparental. By explicitly incorporating mitochondrial mutation and selection in the model, while introducing UPI and mating type mutants, like previous authors, we find that UPI does indeed improve fitness. However, as UPI increases in the population its relative fitness advantage diminishes in a frequency-dependent manner. Critically, the fitness benefits of UPI ‘leak’ into the biparentally reproducing part of the population, limiting the spread of UPI. Only when mating types pre-exist can uniparental mutants become associated with them, leading to a population with strict UPI. We discuss how our findings relate to previous analyses and to the patterns actually seen in protists.

## 2. Model outline

**Figure 1.**
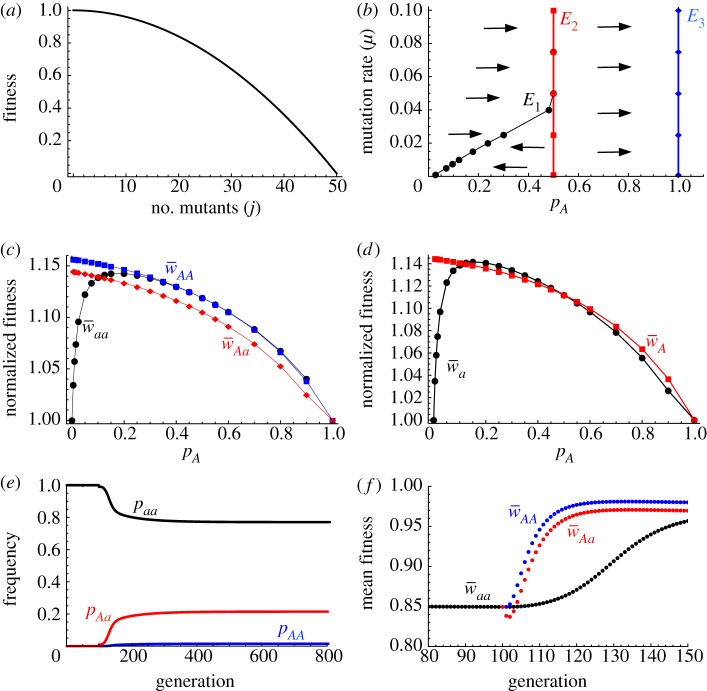
(*a*) Concave fitness curve given by equation (2.1) for *M* = 50. (*b*) Schematic representation of the three equilibria for *M* = 50 and varying *μ*, showing *E*_1_ (black), *E*_2_ (red) and *E*_3_ (blue). Normalized mean fitness (mean fitness divided by mean BPI fitness) of (*c*) genotypes 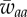 (black line); 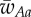 (red line) and 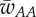 (blue line), and (*d*) genes 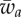 (black line) and 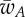 (red line), for fixed values of *p_A_*. (*e*) Trajectories of genotype frequencies through time and (*f*) mean genotype fitness (scaled to maximal fitness of unity for no mitochondrial mutants), from an initial frequency *p_A_* = 0.01 at generation 100. Parameter values: *M* = 50, *μ* = 0.01.

The core model is based on a simple life cycle for an infinite population of diploid, unicellular organisms (see the electronic supplementary material, section A for details of life cycle, derived from [14]). These undergo clonal expansion during which they are subject to mutation and selection. We do not explicitly model this, but for simplicity impose mutation (step one) followed by selection (step two). Each cell contains a fixed number *M* of mitochondria that can be wild-type or mutant resulting in *M* + 1 possible mitochondrial states. The wild-type mitochondria in each cell mutate independently with probability *μ* (back mutation is initially ignored). After mutation, selection changes the relative frequency of each mitochondrial state. Fitness is defined as a concave function of the number of mitochondrial mutations [14],2.1
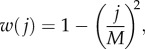
where *j* is the number of mutant mitochondria in the cell (*j* ∈ 0, 1, … , *M*; figure 1*a*). As a cell contains many mitochondria, small numbers of mitochondrial mutations are likely to have minor fitness effects, as suggested by the high threshold of mitochondrial mutations required to cause a significant decline in oxidative phosphorylation [19]. Fitness decline should then be sharper as the mutation load increases, as is indeed evident in many mitochondrial diseases [20]. This justifies the choice of a quadratic fitness function in equation (2.1). We also considered the impact of using a convex fitness function (see the electronic supplementary material, section B1).

Following selection, surviving cells undergo meiosis (step three). The cell's population of mitochondria is doubled to 2*M* and then reduced through two cell divisions to produce four haploid gametes, each with *M*/2 mitochondria. At each meiotic cell division, the mitochondrial genotypes of the parent cell are randomly segregated between the two daughter cells, as is indeed the case in mitotic divisions in eukaryotes (i.e. sampling without replacement) [21]. Gametes then randomly fuse with each other to form the next generation of cells (step four).

We explicitly model the evolution of mitochondrial inheritance by assuming nuclear control through a single locus. Gametes with the wild-type allele *a* cause mitochondria to be inherited biparentally (BPI) when they fuse with other *a* gametes. Gametes with the mutant *A* allele pass on their mitochondria uniparentally (UPI) when they fuse with an *a* gamete, by excluding the mitochondria from the *a* gamete. We define fusions between two *A* gametes as having BPI of mitochondria. We also modelled the alternative assumptions that *A × A* fusions are inviable [12] or result in UPI [13] (see the electronic supplementary material, section B1).

We consider the invasion of the *A* allele into a BPI population of the *a* allele. The *A* allele is introduced at 1%, and then frequency change is tracked through numerical simulations to define equilibria and stability (we assumed no other mutations between *a* and *A* gametes). The rate of UPI is maximized under these assumptions when the frequency of the *A* allele reaches *p_A_* = 0.5. We make small modifications to this basic model, to examine the spread of selfish mutants and the benefits of mitonuclear coadaptation. Note that we assume gamete control of mitochondrial inheritance rather than the diploid parental cell, as this simplifies the dynamics and is true of protists such as *Chlamydomonas* [22]. However, the life cycle is idealized and not intended to replicate any particular protist. A detailed mathematical derivation of the equations used in our simulation is given in the electronic supplementary material, section A. The code was written in C and can be accessed here: https://github.com/UCL/SexesProceedings.

## 3. Results

### (a) Mitochondrial mutation pressure

There are three equilibria 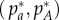: *E*_1_ = (1 − *c*, *c*) where 0 < *c* < 0.5, is stable and reached when the *A* mutant is introduced into a population fixed for the *a* allele (*p_a_* = 1); *E*_2_ = (0.5,0.5) is generally not attractive; and *E*_3_ = (0,1) cannot be invaded by the *a* allele (figure 1*b*). Hence, the uniparental *A* mutant was favoured and spreads in a population that has BPI of mitochondria but only to a polymorphic equilibrium with 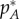 generally less than 0.5. The wild-type allele *a* did not invade a population fixed for the UPI allele (i.e. *p_A_* = 1) and was eliminated if the initial frequency satisfied *p_A_* > 0.5 (figure 1*b*).

To understand the forces determining the three equilibria, we plotted the normalized mean fitness of each genotype and gene while forcing the population to remain at a specified frequency of *p_A_* (mitochondria were allowed to evolve; figure 1*c*,*d*). When the *A* allele is first introduced (*p_A_* ≈ 0), its fitness is effectively determined by matings between *A* and *a* gametes which are always uniparental. As UPI increases variation in mitochondrial mutation load [5,14], the *A* allele is associated with more low and high fitness mitotypes. Given a concave fitness curve (equation (2.1) and figure 1*a*), the net effect is an initial decline in fitness of the *A* allele (figure 1*e*,*f*) [23]. But within a small number of generations (approx. 5 when *M* = 50, *μ* = 0.01; figure 1*f*), the *A* allele accumulates a fitness benefit owing to the cumulative removal of mutant mitochondria made possible by heightened mitochondrial variation. We then have 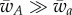, so *p_A_* increases (figure 1*e*).

**Figure 2.**
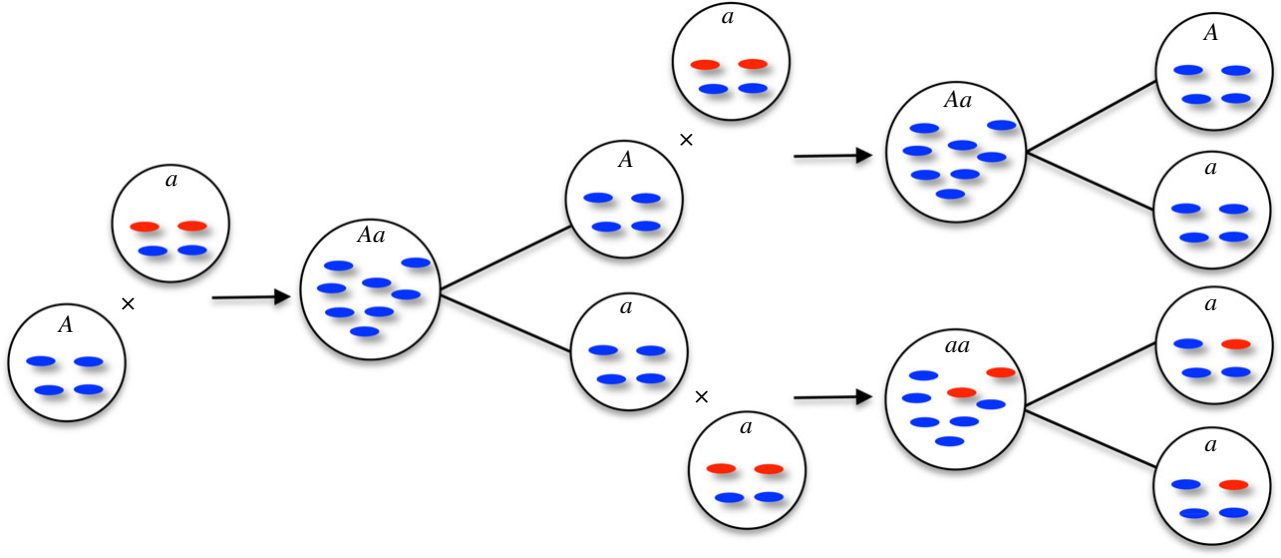
Schematic representation of leakage of UPI benefits to the BPI part of the population. Small circles are gametes, big circles are zygotes. Ovals are mitochondria, blue being wild-type and red mutants. Reading from left to right, when the UPI gene *A* is at low frequency, it becomes associated with fit mitochondria and so *A* gametes are highly likely to carry no mutants (all blue). When a less fit *a* gamete fuses with a fit *A* gamete (first fusion), they produce a mutant-free zygote. In turn, this produces mutant-free *A* and *a* gametes, that are likely to fuse with other unimproved *a* gametes as these are common in the population (second fusion). Even though the resulting *a* × *a* zygotes have BPI, they have lower mutational load than typical *a*×*a* fusions, and produce fitter *a* gametes because of the leakage of improved mitotypes from *A* gametes in *Aa* individuals. Further leakage over many generations leads to the cumulative improvement of mitotypes in the *a* population.

**Figure 3.**
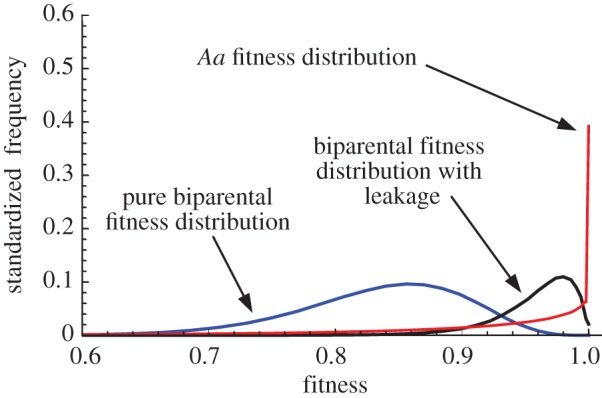
Fitness distribution of a population with strict biparental inheritance when *p_A_* = 0 (blue) showing a low mean owing to the accumulation of mitochondrial mutations. By contrast, at the equilibrium *E*_1_ (*p_A_* = 0.12), the fitness distribution of *Aa* individuals (red) with uniparental inheritance has many individuals having very high fitness (greater than 0.95) owing to repeated cleansing of mitochondrial mutations, although with high variance (*AA* individuals have a similar pattern, not shown). Leakage of this benefit can be seen in the fitness distribution of *aa* individuals (black), calculated at the equilibrium *E*_1_. Parameter values: *M* = 50, *μ* = 0.01.

‘Leakage’ of improved mitochondria is then a key factor that limits the spread of UPI and the *A* allele. When *A* and *a* gametes fuse, the *Aa* zygote inherits the cumulative high fitness mitochondria generated by UPI present in the *A* population. Surviving *Aa* zygotes ultimately produce new gametes, both *A* and *a*. This allows the improved mitochondria to *‘*leak’ into the *a* population (figure 2). Initially, this effect is weak, as *a* × *A* fusions are rare compared with *a* × *a* fusions. But as the *A* allele spreads, leakage becomes more significant and 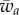 undergoes a sharp rise (figure 1*d*). Thus, the presence of *A* gametes in a population to some degree ‘cleans up’ the mitochondria of the population as a whole, which can be seen in the improved fitness distribution of *aa* as well as *Aa* individuals (figure 3). So as the *A* allele spreads, the relative fitness advantage of UPI declines.

In addition, as *p_A_* increases, *A* × *A* matings become more common. In our model, these have BPI, thereby reducing the rate at which mutant mitochondria are removed. The combination of the short-term disadvantage to *A* of increased variance owing to UPI and the reduction in cleansing of mutations because of *A* × *A* fusions, result in a reduction of 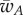 as *p_A_* increases (figure 1*c*,*d*). This, along with the increasing value of 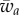 owing to leakage, leads to the polymorphic equilbrium at *E*_1_ (i.e. 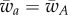). The polymorphic equilibrium *E*_1_ is stable because moving towards *E*_2_ (higher *p_A_*) heightens leakage of improved mitochondria, and therefore further increases the fitness for 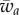 relative to 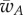 (figure 1*d*).

The second stable equilibrium, *E*_3_, occurs when the *A* mutation is fixed (*p_A_* = 1; figure 1*b*). It might seem paradoxical that this is not invaded by the *a* allele, as this causes UPI of mitochondria. However, the *a* allele is the alternate uniparental pattern of ‘kill your own mitochondria’ rather than ‘kill your partner's mitochondria’ [10]. In this case, the *a* allele does not invade because there is no cumulative purging of mitochondrial mutations (as the *a* allele never passes on its mitochondria, so any benefits of UPI are lost). When *p_A_* is large, any improved mitochondria associated with *a* gametes are lost in the following generation, as mitochondria are only inherited from the *A* gamete, which carries the unimproved biparental mitochondrial state. In addition, as noted above, *A* × *a* fusions increase variation in mitochondria mutation load which in the short-term produces a net fitness disadvantage for the *a* allele (figure 1*f*). These considerations also explain why the equilibrium at *E*_2_ (i.e. *p_A_* = *p_a_*) is unstable. If *p_A_* is slightly lower than *p_a_*, selection drives the population to *E*_1_, and if *p_A_* is slightly higher than *p_a_*, selection drives the population to *E*_3_.

Our results show that alleles causing UPI are subject to frequency-dependent selection. They typically invade to reach an intermediate value (*p_A_* = 0.1–0.2, UPI rate 18–32%, given *μ* = 0.01; figure 1*b*). Only when the mitochondrial mutation rate (*μ*) and number of mitochondria (*M*) are very large, does *p_A_* → 0.5 (i.e. *E*_1_ merges with *E*_2_; figure 1*b*). At these high values, fitness is considerably reduced for both uni- and biparental zygotes (for example, when *M* = 100 and *μ* = 0.1, *E*_1_ merges with *E*_2_ and we have (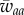, 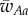, 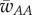) = (0.447, 0.440, 0.447)) (see the electronic supplementary material, section B1). These high values may seem somewhat implausible but are not unreasonable in certain cases (see Conclusions).

We repeated the analysis above using the assumption that *A* × *A* matings are uniparental, with the transmitting role randomly assigned to one of the partners [13]. In this case, the fitness of *A* is fixed and independent of *p_A_*—as all matings involving *A* gametes are uniparental. Nevertheless, fitness benefits still leak from *A* to *a* gametes and an intermediate frequency of *A* is sufficient to ensure that 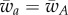, resulting in a polymorphic equilibrium equivalent to *E*_1_. Further complexities related to the existence of *E*_2_ and the stability of *E*_3_ are discussed in the appendix (see the electronic supplementary material, section B1).

We repeated the above analysis using a convex fitness curve. A fitness curve of this nature is hard to justify in unicellular organisms as it predicts that cell fitness should decrease sharply with the accumulation of only very few mutations. If that were the case, there would be a much greater benefit when moving from BPI to UPI, with *E*_1_ and *E*_2_ merging at lower values of *M* and *μ* (see the electronic supplementary material, section B1). The third equilibrium *E*_3_ (i.e. at *p_A_* = 1) also exists but is unstable. Further analysis of the complexities of a convex fitness assumption is included in the electronic supplementary material, section B1.

### (b) Selfish mitochondrial mutants

The selfish conflict theory [8–13] predicts that UPI evolved to protect against the spread of mutant mitochondria that are fast replicators. To explicitly implement this in our model, we included a sampling step after mutation and before selection. In this step, we sample with replacement given that mutant mitochondria have a relative advantage 1 + *k* (so the probability of sampling a mutant mitochondrion is *x*(1 + *k*)/*M* + *xk* given a cell with *x* mutant mitochondria, see the electronic supplementary material, figure S5).

In this case, a similar pattern of three equilibria is found. The value of *p_A_* at equilibrium *E*_1_ increases with *k* (the replicative advantage). Above a threshold value of *k*, *p_A_* becomes equal to 0.5 and equilibria *E*_1_ and *E*_2_ merge (see the electronic supplementary material, figure S6). A replicative advantage for mutant mitochondria is equivalent to increasing *M* or *μ*, favouring a higher frequency of UPI. Thus, selfish mitochondrial mutants increase the spread of UPI. Once again, the third equilibrium *E*_3_ (i.e. *p_A_* = 1) still exists and is stable for the reasons discussed in the previous section.

### (c) Mitonuclear coadaptation

We model mitonuclear coadaptation by using a previous formulation [14], where a gene in the nucleus interacts with a gene in the mitochondria. Both genes have two allelic states, 0 and 1. There are thus three diploid nuclear genotypes (00), (01), (11) and *M* + 1 mitochondrial states. States 0 and 1 in the mitochondria no longer represent wild-type and mutant, respectively, but are either matched or unmatched mitochondria with respect to the nucleus. The impact of each allele on cell fitness depends on this interaction between the nucleus and mitochondria,3.1
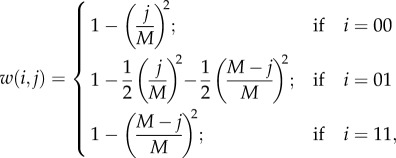
where *i* is the nuclear state and *j* the mitochondrial state [14]. We also modify the mutation step in the life cycle so nuclear and mitochondrial genes mutate with probabilities *ν* and *μ*, respectively (assuming 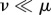), and equally in both directions, 0 ↔ 1.

The complexities of this model have been discussed before in a non-evolutionary context [14]. Apropos the evolutionary discussion here, a similar pattern of three equilibria is found once again. The nucleus converges to one of the homozygote states (00 or 11) which the mitochondria largely match (mainly 0 or 1). At this coadapted equilibrium, mitochondria can again be thought of as wild-type (matching the nucleus) or mutant (in disagreement with the nucleus), which explains why the two models yield similar results. In general, the value of *p_A_* at equilibrium *E*_1_ is lower for similar mitochondria mutation rates and *E*_1_ did not merge with *E*_2_ even under increased *M* or *μ*. This reflects the symmetry of mutation between the mitochondria states (0 and 1) which mutate between each other with the same probability, generating matched (from unmatched) as well as unmatched (from matched) mutants. Because the nuclear alleles can adapt to mitochondrial mutations, and vice versa, the effective mitochondrial mutation rate *μ* is lower than in the mutation accumulation model, explaining why *p_A_* is lower at equilibrium.

A further complication is that external factors, for example environmental pressures, could result in periodic switching of the dominant mitonuclear state (0 or 1). For example, mitochondria adapt to both temperature and diet, and fluctuations in either might in principle undermine fitness [24,25]. We implemented this by periodically imposing a cost to the dominant state, forcing it to switch (see the electronic supplementary material, section B3). These switches favoured higher values of *p_A_*, closer to 0.5. Higher levels were favoured as UPI aided faster switching to better-adapted mitonuclear states. However, once a general state of mitonuclear coadaptation had been achieved, *p_A_* returned towards the equilibrium at *E*_1_ (see the electronic supplementary material, figure S7)*.* Only under strong and frequent switching did the frequency of the uniparental allele (*A*) rise significantly, but even then never to *p_A_* = 0.5.

### (d) Mating types

In the analyses above, we consider the spread of a mutation inducing UPI. However, the fact that *A* × *A* fusions are still possible means that BPI cannot be eliminated unless *A* and *a* become associated with self-incompatible mating types, as is the case in many unicellular eukaryotes [3]. We modelled this by considering that a further nuclear locus controls self-compatibility, denoted by the mating type index *m* (*m* ∈ 1, 2,…). Gametes can fuse with anyone but self (e.g. *a*_1_ will not fuse with *a*_1_/*A*_1_ but can fuse with *a*_2_/*A*_2_ and so forth). The presence of mating types potentially allows complete UPI if the *A* and *a* alleles are associated with different mating types.

**Figure 4.**
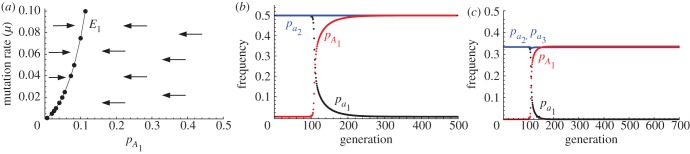
Equilibria with mating types. (*a*) When the uniparental inheritance mutation is linked to a mating type allele (*A*_1_) that controls self-compatibility, there is an equilibrium at *E*_1_ with 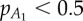, but no equilibria at *E*_2_ or *E*_3_ (*μ* = 0.01 and *M* = 50, for comparison with figure 1*a*). (*b*) Change in gene frequency across time (generation), from introduction of the uniparental inheritance mutation *A*_1_ allele (initial frequency 10^−2^ at generation 100) into a population with biparental inheritance but two pre-existing mating types until stability is reached at equilibrium. With high values of *M* = 100 and *μ* = 0.1, *a*_1_ is replaced by *A*_1_ and the equilibrium population has strict uniparental inheritance. (*c*) With more mating types (three in this case), *a*_1_ is again replaced by *A*_1_, but there is no reduction in the number of mating type (*M* = 100, *μ* = 0.1).

We first introduce *A*_1_ into a population fixed for *a* (i.e. *p_a_* = 1), assuming that the mating type index is linked to the UPI modifier. The frequency of *A*_1_ increases but only to an intermediate point equivalent to *E*_1_ (figure 4*a*). This is for similar reasons that *A* reaches an intermediate frequency in the previous section; in particular that the benefits of UPI leak into the *a* population. A notable distinction is that *A*_1_ × *A*_1_ fusions are impossible, so *A*_1_ has a mating rate disadvantage. This results in 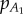 having a slightly lower value at *E*_1_.

Adding a further mating type allele *a*_2_ could be advantageous, as *A*_1_ × *a*_2_ fusions are exclusively UPI. However, the *a*_2_ allele decreases monotonically in a population at *E*_1_ (or for other non-zero values of 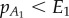). The *a*_2_ allele does not spread because it does not improve fitness beyond what is already achieved through *A*_1_ × *a* fusions but has a slight mating rate disadvantage (as it is self-incompatible).

We then introduce *A*_1_ into a population with two pre-existing mating types *a*_1_ and *a*_2_ (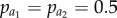). Again *A*_1_ spreads, this time at the expense of *a*_1_ alone, to a stable state corresponding to *E*_1_, with the population at equilibrium made up of *A*_1_*a*_2_ and *a*_1_*a*_2_ individuals. For the same conditions that equilibrium *E*_1_ merged with *E*_2_ without mating types (i.e. high *M* and *μ*, selfish mutations or a convex fitness curve), *A*_1_ displaces *a*_1_ altogether, leaving a population with two mating types with strict UPI (figure 4*b*). If the UPI mutation (*A*_1_) invades a population with three, four or more mating types, we find that equilibrium frequency 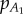 is reduced as the reciprocal of the number of mating types (figure 4*c*). There is no collapse to two mating types. All the analyses above were repeated assuming full recombination between the mitochondrial inheritance locus and the mating-type locus. When full recombination was assumed, the mutant imposing UPI (*A*) spreads to a similar frequency as before. However, in this case, each mating type becomes associated with both UPI (*A*) and BPI (*a*) alleles. So full UPI is only possible with tight linkage (see the electronic supplementary material, section B4).

## 4. Conclusion

A number of experimental and theoretical analyses indicate that UPI of mitochondria can improve fitness and has led to the suggestion that this force underlies the evolution of two mating types, leading to anisogamy and the evolution of true sexes [8–14,26,27]. Our modelling confirms that UPI does indeed improve fitness. This holds when UPI reduces mutation load, limits the proliferation of selfish mutants or improves mitonuclear coadaptation. But our explicit consideration of mitochondrial evolution, with UPI or BPI and mating types, shows that there are real limits to what is possible.

We show that the fitness benefits arising from UPI are acquired cumulatively, over multiple generations. In the short-term, increased variation is detrimental, as many low fitness variants are generated. It is only after selection has had time to remove these less fit individuals that better mitochondrial adaptation builds up. Crucially, during the spread of a mutant that induces UPI, the fitness benefits of UPI inevitably leak through the population. The BPI part of the population benefits from regular infusions of fit mitochondria from UPI gametes, and leakage increases with the frequency of the UPI mutant. This makes the fitness advantage of UPI frequency dependent, and generally limits the spread and fixation of UPI mutants.

We find that UPI itself cannot drive the evolution of two distinct mating types. Others have also expressed uncertainty as to whether mating types can be understood as a consequence of UPI, but empirical data are conflicting and do not unambiguously support or refute the UPI hypothesis [4,21,26,28,29]. Nonetheless, it seems likely that mating types existed before the evolution of UPI [4,28,30–33]. Only when we assumed the pre-existence of two mating types did we find conditions that can drive UPI to fixation, in particular, high mutation rates, large mitochondrial numbers or selfish mutants. Very high mtDNA numbers and mutation rates are possible. For example, the amoeba *Pelomyxa carolinensis* has as many as 300 000 mitochondria [34]. Likewise, the mitochondrial mutation rate can be extremely high—estimates for petite mutants in yeast are orders of magnitude higher than the nuclear rate [35]. We also find that for UPI to go to fixation, it is necessary that the gene for mitochondrial inheritance occurs in tight linkage with the mating type locus, although some UPI is possible without linkage. We finally observe that the invasion of a UPI mutant cannot reduce the number of mating types in a population that already possesses more than two mating types.

Our results contrast with those reported from previous modelling work [10–12,14]. In large part, this is owing to their unrealistic assumption of a fixed cost for BPI caused by cytoplasmic mixing. The assumption that cells suffer the same cost independently of the number of mutants they carry totally alters the dynamics. Our work shows that it is important to consider the frequency-dependent interplay of costs and benefits associated with each mode of inheritance which naturally leads to the emergence of intermediate values of UPI and BPI. Our results echo those of Hastings [13], who explicitly modelled mitochondrial evolution. However, the significance of leakage was not studied or discussed in any detail, leading to (in our view) an inappropriate weight being placed on UPI as the motor force for the evolution of two mating types and anisogamy [13].

Like previous authors [10,13,36], we found that a UPI modifier which kills its own mitochondria will not spread. The major problem is that such a modifier cannot become associated with the fit mitotypes and so the potential benefits gained though cleansing of mitochondrial mutants are always lost in the following generation. An exception to this rule is when selection on mitochondrial mutants follows a convex curve. Then UPI spreads because higher variance in mitochondrial mutation load is favoured each generation (see the electronic supplementary material, section B1). However, a convex fitness relationship seems unlikely to be a general feature of mitochondria mutants or heteroplasmy, so the relevance of this result may be limited. This is certainly the case in mitochondrial diseases, where mutant load must be greater than about 40% before any symptoms become apparent, and there is no reason to suppose that single-celled organisms are any different in this regard [20].

A related issue is the possibility of negative epistatic effects arising from the mixing and interaction of different mitotypes [27,37]. We have not explicitly modelled this here. But our results suggest that hybridization between populations with different, incompatible mitotypes will evolve towards a homoplasmic state, as was found experimentally by Sharpley *et al*. [27]. This is equivalent to our formulation of mitonuclear coadaptation, where genes in the nucleus and mitochondria need to match in order for efficient function. However, as mentioned above, we did not find that this qualitatively altered the frequency-dependent outcome of selection for UPI. Finally, we should mention that unlike other authors, we impose no additional cost for UPI (e.g. related to the need for amplification of a smaller cytoplasm [10]). In that sense, our model is conservative and even tighter conditions for the spread of UPI and mating types would be expected had those costs been implemented.

Our findings suggest a continuum of UPI levels is possible depending on the energetic demands (number of mitochondria), mutation rates and nature of mutations (selfish or not). This prediction is consistent with a number of empirical observations: the prevalence of some degree of UPI in unicellular eukaryotes [38]; the presence of a mixture of maternal or paternal UPI as well as biparental zygotes in some unicellular organisms, slime moulds and plants [3,39]; the persistence of BPI in organisms with two mating types, for example yeast [40]; numerous distinct mechanisms of generating UPI (implying multiple origins and fluctuating selection) [4,38]; and tight linkage of mitochondrial inheritance and mating type loci associated with apparently strict UPI in some protists such as *Chlamydomonas reinhardtii* and *Chlamydomonas smithii* [41,42]. But better investigations of the natural variation in rates of UPI and associations with mating types in protists are needed, as are more studies of mitochondrial number and properties. Mitochondrial number, size and behaviour differs between cells of the same [43] and closely related species [44], potentially accounting for differences in the spread of UPI in protists.

One matter that remains to be addressed is the importance of UPI and mating types in the evolution of anisogamy. Future work needs to examine the role of mitochondrial fitness in the tight linkage of UPI with the germline/soma distinction of multicellular organisms.
